# Supplementary material for: Women’s strategies for navigating a healthy sex life post-sexual trauma
Source: PLoS One. 2023 Sep 6;18(9):e0291011. doi: 10.1371/journal.pone.0291011 (PMC10482293; doi:10.1371/journal.pone.0291011)
Supplement: S1 Table — (DOCX) [file pone.0291011.s001.docx]

**Table 1.** Representative Quotes for Each Theme of Effective and Ineffective Strategies

| Theme | Representative Quotes |
| --- | --- |
| Effective Strategies |  |
| Social Support | So one is the circles that I surround myself with. . . giving me the freedom or space to grow and to think about the things that I've experienced in my own life through hearing other people's situations. An example of what I'm talking about specifically is I'm involved in a women's organization. We meet and support each other through either current or past situations that we have encountered in our lives and being a part of that group has been very healing for me. *-Holly, 25-year-old white participant*  So, people with similar experiences and talking to them has been huge for me and talking to people who don't have similar experiences but are understanding. *-Ariel, 37-year-old white participant*  My mother was a huge support. She was there with me at the police station. And currently I just have a group of these amazing, world changing women in my life who some had, like one is a volunteer for the Rape Crisis Center here. I have a bunch of friends who were seeing nurses. So like I just have a lot of, I have a lot of support especially in my current community. *-Clare, 42-year-old Jewish participant*  I would say that actually working in sexual assault prevention has done just amazing things for me, because I do talk about it all the time . . . just being able to use my experience to try and help others ... And, unfortunately, my help is a lot of times not the prevention I would like it to be, but a lot of times students, after the fact, will come and talk to me. At least I can get them to therapy or get them to someone who can really help them with what they need. That I feel like heals my soul a little bit. *-Noelle, 42-year-old white participant*  Yeah. Just trying to be more open with my female friends, because I realized that this is not uncommon and that several of my friends have also had traumatic experiences. So, just being able to try and find friends who have been through similar experiences, which you don't know until you talk about it with them. *-Corrine, 24-year-old white participant*  Listening to other people's stories . . . other people's writing, talking, where it's journal, I mentioned like a blog, things like that are really validating because common themes to hear about. You say “oh, they felt that, I felt that too” it feels more like your feelings make sense. If they didn't make sense to you at the time. *-Ariel, 37-year-old white participant* |
|  | I feel wildly lucky to have a huge network of friends, especially friends that have sexual trauma histories. At any time, there are at least four women that I can step out and call and go on a walk on the phone like this and get that support. Some of the people that I care about aren't connected to big networks like this, and wow, that would be so hard to be dependent on your primary male partner for your emotional support. *-Mel, 28-year-old white participant*  Making sure I surrounded myself with supportive people, because it's funny. When you talk about these things, there are definitely different reactions you get, some of which are amazing and just genuine and wonderful, and then others that just make you think, wow, I can't believe I thought that we were friends. Honestly, that would be my best [inaudible] therapy, supportive people. Get your tribe. *-Noelle, 42-year-old white participant*  I would never ever listen to myself but being able to identify people who are safe for you and then being able to identify people who aren't safe for you and increasing the number of people who are safe for you in your life. *-Alicia, 25-year-old white participant* |
| Communication | I think probably actually just talking about it, both with friends and with my partners has been, particularly with my current partner, it's been really helpful in my healing process to be able to talk very frankly and openly. *-Margo, 29-year-old white participant*  I think it's so important. I feel like a lot of times we don't talk about this stuff enough and how prevalent it really is. . . so I think the more that we talk about, maybe they'll open up a little bit more about things that have happened to them. *-Callie, 24-year-old white participant*  I guess just the big key is communication both with, also having at least one person outside a relationship, outside any kind of sexual relationship, who you just trust unconditionally and is there throughout different relationships, and breakups, and things, who you can just always talk to. It's immeasurably valuable. I know a lot of people don't. *-Olivia, 26-year-old white participant*  Definitely like learning how to use my words better has been a really big thing because the commonality with both incidents was I could have said no and I didn't. So, getting more comfortable with using my words is a big thing for me. *-Rebekah, 37-year-old white participant* |
|  | In my current relationship, at least we've had many healthy issues discussions of boundaries and consent and things like that, because that's really important to me. *-Corrine, 24-year-old white participant*  As far as sexual coping strategies, I mean communication is always kind of the number one thing. And this is also kind of a strange thing, but like stopping sex pretty often at the beginning, at different points . . . just to make sure that I can, which actually, I mean it wasn't fun for him, but it really helped me a lot. *-Cindy, 26-year-old white participant*  I try to be very, very clear, and I try to express as much as possible if I do feel something negative, I try to be clear because then that's when I start to shut down and if I don't tell him, then he's not going to know, so I have to just be very clear. The thing that helps me the most is communication. *-Stef, 26-year-old Black participant* |
| Empowerment | As far as self help ... let's see. It keeps bouncing back to the self empowerment, so ways that I make myself feel empowered. I have specific self care days. That is very important for feeling good, empowered, and like I'm in control of my life. *-Abby, 20-year-old white Hispanic participant*  I've made a really big point in my life to not define myself by my sexual traumas. . . It was a matter of finding empowerment in my sexuality, rather than letting these boys have the power. For me, it was becoming very interested in sexuality as a whole, and not whispering when I said the word 'sex', but being very confident about it. Like, "Sure, I have sex. That's fine" and owning it. *-Katarina, 22-year-old white participant*  I felt like I love myself and the fact that this happened to me doesn't change that or I love the person that I've become and this is part of me becoming the person that I am and so I could move on from it. *-Sarah, 24-year-old white Filipino participant*  And I think, for me, a big part of what has worked has been focusing on building positive body image and building comfort in my own self. *-Nicole, 42-year-old white participant* |
|  | Something that helped me a surprising amount was coming to the realization that my ex-boyfriend and I were BOTH victims of rape culture and toxic societal expectations, which allowed us to be complicit in the event. He wasn’t a monster or a villain, he was just a stupid kid who thought that that behavior was normal. Realizing this allowed me to let go of my pent up anger toward him, reach out to him and learn that he too had grown to become a better person and matured past that toxic culture. I was then able to turn that energy into something positive, focusing on changing that culture for our young men and women through education. That’s part of why open communication is so important to me now, because we can talk about how society’s expectations affect us and how we can be aware of/work around these. *-Olivia, 26-year-old white participant*  So when I realized, "Okay. You didn't make this up. No, I didn't make this up. There's nothing wrong with me. This is something that happened to me." That was the really important step for me. Just realizing that there was nothing wrong with me. I didn't do anything wrong, that this was something that happened to me basically *-Sarah, 24-year-old white Filipino participant*  I do my best to be open about it. I've learned that just looking at it as something that has happened in the past and not something that I identify with helps a lot with that. *-Lucy, 25-year-old white participant* |
| Self-Care | For me, meditation and yoga and those kinds of grounding activities ultimately, although not in a sexual context, translate sexually for me because then I feel more in control of my body and safer, I guess, because I'm used to being present in being myself anyway. *-Nicole, 42-year-old white participant*  I think just general things I have done to benefit myself in a very personal way. Like, doing better in school, or going to the gym, or the typical New Year's resolution things that people talk about to make themselves feel better about themselves. And I think just things that have really benefited my self-esteem have been a big, big thing for sexual healing, as it were. *-Jess, 26-year-old AfroLatina participant*  Just general kind of mental health and anxiety management techniques I find really helpful, and it may not just in this situation and many situations, like breathing, and muscle relaxation, and visualization techniques. *-Olivia, 26-year-old white participant*  I do a lot of meditation now. I feel like that's really helped me in my relationship when things are getting a little bit stressful or we're disagreeing on something. Just having a 10 minute guided meditation really just gives you a little bit clearer of a mindset. Exercising I think has really helped me. *-Callie, 24-year-old white participant*  And I would say it's like a process. I mean I like yoga and things like that, but I wouldn't say that's something that necessarily was a good driver for me. It was more just like exercise. I mean I wouldn't say that was like super sexually, but making myself feel better about myself physically made me more sexually comfortable. *-Allie, 22-year-old white participant* |
|  | I have also found therapy in journaling. I think that just is a helpful kind of low cost way to kind of get that out of the box that's in our head and get them out on paper and so they're not necessarily there suffocating us. *-Jane, 33-year-old white participant*  And actually, one of the most useful exercises that I've ever done for that is I wrote this story of my trauma, all of them, I just wrote them all down. Basically, everything that I was hurt by in my entire life and the things that were keeping me in the state of victim hood which perpetuates on trauma. I wrote down my entire story and then I went back and I said for each time I was traumatized, what have I learned from it? That I really like about myself now. You know, for instance, I was molested by a family member at a young age and because of that I learned really strong boundaries. *-Alicia, 25-year-old white participant*  So painting, and poetry, or just journaling, or just your own forms of expression about it. I've never made a public post about it where some people do that didn't appeal to me. But just your own private journaling or my art that expresses it and other things like that I think are usually helpful. *-Ariel, 37-year-old white participant*  Yeah. I paint. That's one of the biggest things that I do and I write. I write a lot of letters to people that I never send. Writing is something that helps and then painting, for sure. *-Cait, 22-year-old white participant*  I write in a journal, and that helps a lot because I can write down what I'm feeling in the moment, and then I can go back a little bit later and say "Oh. I saw this is a different way yesterday than I see it today, and today I can come back to it and kind of work on it from a different angle. *-Mindy, 26-year-old white participant*  For me, meditation and yoga and those kinds of grounding activities ultimately, although not in a sexual context, translate sexually for me because then I feel more in control of my body and safer, I guess, because I'm used to being present in being myself anyway. *-Nicole, 42-year-old white participant* |
| Therapy | I mean I definitely have tried to go to a lot of therapy and I think finding a good therapist has been really impactful for me. I think it's like a lot of people say, Oh yeah I go to therapy its great but they don't really tell people how to do that. The way this works, for me at least, is to book special appointments with five different people and then see which person clicks . . . I always look for someone who has experience with sexual trauma. *-Alicia, 25-year-old white participant*  I am so lucky. I, from the very beginning, this happened to me right as rape crisis centers were beginning to exist. So I've been in therapy, cognitive behavioral therapy. Pretty much continuously ever since. *-Olivia, 26-year-old white participant*  I had a therapist at the time. But other than that, I didn't really talk to other people about what I was going through so it was really just the support that I had through therapy. *-Jane, 33-year-old white participant*  After the trauma when I was 11, I was court ordered to see a therapist. I am a huge advocate of therapy. If people are into that, I think that therapy is a really great method for coping. *-Katarina, 22-year-old white participant*  A whole lot of people in my life don't really know about what happened. I'm pretty quiet about that stuff. I went through a lot of therapy, so I feel just like cognitive behavioral therapy and like DBT skills, they really come in handy. *-Callie, 24-year-old white participant* |
| Ineffective Strategies |  |
| Casual sex | Or just having random sexual encounters with people to try and ... I mean, not random, but sexual encounters that maybe you wouldn't have otherwise, just to feel something. *-Noelle, 42-year-old white participant*  After the incident, and I had never been a overly promiscuous person. I was also fairly young. But then after that, I went off the rails with it, and I was like, "I'm going to just sleep with everybody, because nothing matters and none of this is important. And I don't value my body. . . Like, I was very much in a mindset of my only worth as a person is in my ability to have sex, because the incident did happen at a time when I was very socially isolated and failing academically. And I didn't have the best relationship with my family. And everything felt like it was crashing down around me, and this was the one thing I felt like I potentially had value in, where I was like, "I can connect with people this way," or, "Be praised this way," or, "Feel good about myself this way." It was the only way that I tried to cope, initially, being very hyper-sexual, almost. It was almost manic, in a way, when I think about it. But I found that that was very much not a helpful strategy. *-Jess, 26-year-old AfroLatina participant* |
| Ineffective Communication | I always found it really frustrating when someone tries to say like, "Yeah, I get it," or "I know what you're going through." And I'm like, "No, you don't. You don't know the situation." *-Allie, 22-year-old white participant*  Well, I think one thing that I will mention that in hindsight didn't work so well is talking to too many people. So, a lot of it surfaced at that point. And so, it was like, just a floodgate opened, and I remember just talking to so many people. It was almost like I needed a megaphone. Like everybody needs to hear about this. And that was my coping at the time, because that's how I tend to process other things. I can see that it not only was it overly exposing and too vulnerable for me, but it also muddied things. Because naturally you share with people, unless they're a trauma counselor, they're shocked. And then they want to try and fix it. And so, the responses I would get would either be shock and awe, or weird advice. Which then sometimes I would actually take, or I wouldn't, or it would be confusing. So, it was just too much. *-Heather, 42-year-old white participant* |
| Avoidance | I think being, so like coping by dissociation was effective in the moment and not helpful long term. And some of that's like for a little while it was an automatic response, and then it was like a practice plan. I was like, "All right, time to check right out." That was effective in the moment and just also didn't contribute to a fulfilling relationship or to a fulfilling experience of sexual pleasure. *-Margo, 29-year-old white participant*  So, a sexual strategy that I've tried again with my first sexual partner was to like think of literally anything else besides what was happening, and that made me very distracted. It was just not good. So that's one, that's like the first sexual strategy I tried, and it failed. *-Hannah, 21-year-old white participant*  And for months and months I told you I was using like avoidance strategies first. So I finally got to the point where I was like, "I don't want to do this anymore. It can't be like this. I need to have healthy relationships again." *-Allie, 22-year-old white participant*  And this is really not a good coping strategy, it's a bad one. A self-sabotage relationship, so I don't even have to experience intimacy to protect myself in some way, like keep myself safe from emotional, physical, any kind of hurt by sabotaging pretty much any relationship that I would even begin to be a part of. Or even if it was a committed relationship, still finding myself in moments of vulnerability, going back to that sabotaging kind of mechanism. *-Sarah, 24-year-old white Filipino participant.* |
| Substance Use | Other things that I wouldn't recommend, I definitely wouldn't recommend trying to cope with drugs or alcohol. I mean, trust me. I understand the pain that comes with it. But the pain that comes with trauma I should say, but drugs and alcohol aren't going to help resolve those issues. They're not gonna bring a person to a better place. They're just going to mask everything, and I'm not knocking that. Sometimes you just need that. Sometimes you just need a time out from your emotions, but that's definitely not a viable longer-term solution. *-Mindy, 26-year-old white participant*  I would say drinking has not worked. I don't have a drinking problem, I'm not an alcoholic, but I would say my freshman year of college I definitely just drank a lot. *-Mary, 26-year-old white participant*  Drinking heavily did not work. *-Noelle, 42-year-old white participant* |
| Therapy | So I have seen a couple of therapists here and there without too much luck. I mean, I just feel like being a very loud, clear sex worker is not exactly middle class therapist friendly. There are a lot of buttons that you can push and I will leave. *-Mel, 27-year-old white participant*  Yeah. The one thing that was really important to me during the relationships that I had in which I was raped is that I could find a counselor or therapist who was a woman of color and queer. And being on a university campus with a clinic that is really understaffed and underfunded, I didn't find that magical person. *-Justine, 25-year-old white participant*  I guess maybe the EMDR. I don't know if I ... I wouldn't discourage anyone from trying it, but I don't think it worked for me ... I don't know. I just didn't enjoy it, I didn't like it. I didn't like the way I felt afterwards. I did not enjoy that. *-Kate, 37-year-old white participant* |
| Self-Harm | Yeah. So years ago I used to cut. And I know that that was an unhealthy coping mechanism and it scared a lot of people because people equate cutting with suicidal ideation or suicidal behavior, paired with suicidal behavior. And so, I've worked hard to control my cutting urges. I still get urges a couple times a year, but that's different than a couple of times a day, which I had gotten many years ago. *-Mindy, 26-year-old white participant* |
