# Supplementary material for: Women’s strategies for navigating a healthy sex life post-sexual trauma
Source: PLoS One. 2023 Sep 6;18(9):e0291011. doi: 10.1371/journal.pone.0291011 (PMC10482293; doi:10.1371/journal.pone.0291011)
Supplement: Attachment — Submitted filename: Response to Reviewers.docx [file pone.0291011.s002.docx]

Response to Reviewers for PONE-D-23-04051

“Women’s strategies for navigating a healthy sex life post-sexual trauma”

Reviewer 01

I would like to give my feedback on your content. Firstly, it is important to include the demographics of your participants in order to provide vital information in relation to the results obtained. Additionally, it is crucial to include a discussion of the ethical considerations taken into account during your research, as well as your positionality and reflexivity in order to ensure transparency and accountability.

🡪We have revised the paper to include demographic characteristics of our participants in the method section of the paper but also after each of the quotes. And thank you for bringing up positionality and reflexivity – we have added this to the discussion section as suggested and think this improves the richness of the manuscript.

Furthermore, I recommend that you write a separate conclusion for your research, which should summarize your findings and provide future recommendations for further study. It is also important to address any limitations and implications of your study, as this will help to contextualize your results and provide a more comprehensive understanding of your research.

🡪 We have provided a separate conclusion for the research at the end of the paper and it does help to contextualize the results. Thank you for this suggestion. Additionally, we added more explicit limitations and future directions to the discussion of the paper.

I hope these points are helpful in improving the quality of your research.

🡪These points are very helpful in improving the quality of our research and we appreciate that you took the time to review our manuscript.

Reviewer 02

Authors did not follow the Prescribed format for Quotations in Themes like Long quotations in the Vancouver Style are held to have 40 words or more. These are laid out in a separate paragraph of text and indented clearly from the left margin. No inverted commas/quotation marks are included while Short quotations are held to be less than 40 words in the Vancouver Style required double space and inverted commas so please check this mistake and rectify it.\

🡪 Thank you for taking the time to review the paper and for this suggestion, it helps with flow of the paper to have it laid out in this way. We have made this change throughout.
